# Shape Matters in Magnetic-Field-Assisted Assembly of Prolate Colloids

**DOI:** 10.1021/acsnano.1c09208

**Published:** 2022-02-09

**Authors:** Antara Pal, Carlo Andrea De Filippo, Thiago Ito, Md. Arif Kamal, Andrei V. Petukhov, Cristiano De Michele, Peter Schurtenberger

**Affiliations:** † Division of Physical Chemistry, Department of Chemistry, Lund University, Lund SE-22100, Sweden; ‡ Dipartimento di Scienze, Università degli Studi Roma Tre, Via della Vasca Navale, 84, 00146 Rome, Italy; ¶ Centre Interdisciplinaire de Nanoscience de Marseille (CINaM), CNRS, Aix Marseille University, Campus de Luminy − Case 913, 13288 CEDEX 09 Marseille, France; § Van’t Hoff Laboratory for Physical and Colloid Chemistry, Utrecht University, Utrecht 3584 CH, The Netherlands; ∥ Laboratory of Physical Chemistry, Eindhoven University of Technology, Eindhoven 5600 MB, The Netherlands; ⊥ Department of Physics, Università di Roma La Sapienza, I-00186 Rome, Italy; # Lund Institute of Advanced Neutron and X-ray Science LINXS, Lund University, Lund SE-22370, Sweden

**Keywords:** directed self-assembly, magnetic anisotropic colloids, liquid crystals, small-angle X-ray scattering (SAXS), Monte Carlo (MC) simulation, particle shape-analysis

## Abstract

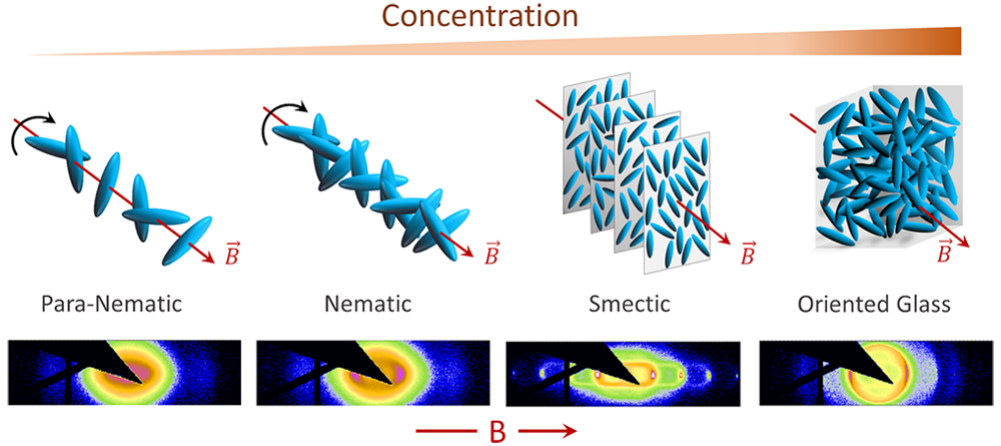

An anisotropic colloidal shape in combination with an externally tunable interaction potential results in a plethora of self-assembled structures with potential applications toward the fabrication of smart materials. Here we present our investigation on the influence of an external magnetic field on the self-assembly of hematite-silica core–shell prolate colloids for two aspect ratios ρ = 2.9 and 3.69. Our study shows a rather counterintuitive but interesting phenomenon, where prolate colloids self-assemble into oblate liquid crystalline (LC) phases. With increasing concentration, particles with smaller ρ reveal a sequence of LC phases involving para-nematic, nematic, smectic, and oriented glass phases. The occurrence of a smectic phase for colloidal ellipsoids has been neither predicted nor reported before. Quantitative shape analysis of the particles together with extensive computer simulations indicate that in addition to ρ, a subtle deviation from the ideal ellipsoidal shape dictates the formation of this unusual sequence of field-induced structures. Particles with ρ = 2.9 exhibit a hybrid shape containing features from both spherocylinders and ellipsoids, which make their self-assembly behavior richer than that observed for either of the “pure” shapes. The shape of the particles with higher ρ matches closely with the ideal ellipsoids, as a result their phase behavior follows the one expected for a “pure” ellipsoidal shape. Using anisotropic building blocks and external fields, our study demonstrates the ramifications of the subtle changes in the particle shape on the field-directed self-assembled structures with externally tunable properties.

## Introduction

Over the past couple of decades the focus of colloid science has gradually witnessed a shift toward understanding the behavior of anisotropic particles; particles having anisotropy either in their shape or interaction potential or both. Anisotropic colloids exhibit a rather complex and rich phase behavior in comparison to their isotropic analogues, which makes them particularly interesting model systems in various areas of condensed matter physics and materials science. The prospect of tuning their self-organization by modifying the anisotropy in their shape in conjunction with the possibility of introducing an anisotropy in the interaction potential using external fields has immensely contributed toward a better understanding, design, and control of self-assembled smart materials.

Depending upon the particle shape and the interaction potential, suspensions of anisotropic colloids manifest different self-assembled structures encompassing *isotropic*, *nematic*,^1−12^*smectic*,^13−15^ and *columnar* phases.^16−20^ One of the most important features that distinguishes these phases from one another is the presence (or absence) of orientational and positional order. In the isotropic phase, the particles possess neither orientational nor positional order, whereas in the nematic phase, there exists a long-range orientational order but an absence of long-range positional order. Smectic and crystal phases are generally characterized by the presence of both long-range orientational as well as positional order.

The use of external electromagnetic fields to manipulate the orientational interactions of anisotropic particles and drive their self-assembly has been in the spotlight for the last couple of years (^7,21−31^ and references therein). The fast and reversible nature of the field-induced dipole–dipole interaction between the particles, as is the case in these systems, makes this bottom-up approach extremely versatile. In view of the ease in manipulating the interaction potential of magnetic particles through an external field, we have in the present study focused our attention toward investigating field-directed self-assembly of ellipsoidal particles with a magnetic core.

Over the past decade, a significant amount of work has been done toward elucidating the theoretical phase diagrams for ellipsoidal particles. These studies predict the existence of isotropic, nematic, and SM2 crystalline phases as a function of concentration and axial ratio.^32−34^ It is interesting to note that so far there are neither any predictions nor any experimental reports for the existence of a smectic phase in case of ellipsoidal particles. This is in stark contrast to the situation encountered, for example, for particles with cylindrical or spherocylindrical shapes.^35,36^ In this article, we have not only revisited these theoretical predictions for ellipsoidal and spherocylindrical particles, but we have, in addition, used an external field to investigate the effect of a partial reduction in the rotational degrees of freedom of the ellipsoids and spherocylinders.

In the case of anisotropic particles, the presence of an external magnetic field results in an alignment of the magnetic moment of the individual particles along the field direction, thereby restricting rotational motion of the particles accordingly. For prolate particles the induced dipolar moments are in general along their long axis. However, this is not the case for prolate particles consisting of a magnetic core that is made up of hematite (α-Fe_2_O_3_) spindles. In this scenario, the particles align with their short axis parallel to the field direction.^37,38^ The explanation of this rather striking behavior rests on the fact that in the case of hematite, the easy axis of magnetization resides within the basal plane of the hematite and is oriented perpendicular to the spindle axis. Consequently, the direction of the induced magnetic moments is perpendicular to the long axes. Although at sufficiently large field strengths, the particles align with their short axis parallel to the magnetic field, they can however still individually rotate around their magnetic moments provided the volume fraction is low enough and interparticle interactions are negligible.^38^ Further, for most systems, the application of an external field also induces additional dipole–dipole interactions, which strongly influence the self-assembly process. In contrast, for the hematite particles used here, the small magnetic core and the canted antiferromagnetic nature of hematite result in a small magnetic moment of the particles. Together with the silica-based shell that further increases the distance between the magnetic cores at contact, the dipolar interaction turns out to be 2 orders of magnitude smaller than *kT*, and the application of the external field only aligns the particles with their short axes being parallel to the field direction.^37−40^

In the present article, we have exploited the aforementioned property of hematite to tune the self-assembly of prolate colloidal particles for two different aspect ratios of ρ = 2.9 and ρ = 3.69 at different concentrations, whose shape closely resembles an ellipse at a first glance. Using gravity to create a sedimentation profile (or concentration gradient) in a dispersion of silica-coated hematite colloids allows us to efficiently sample a large range of the phase space within a single sample. We report that for the particles with the smaller aspect ratio (ρ = 2.9), four different self-assembled structures exist: *para-nematic*, *nematic*, *smectic*, and *oriented glass*, respectively. A quantitative analysis of the SAXS patterns clearly reveals that it is possible to create oblate self-assembled phases with prolate particles through the application of an external field. Astonishingly, our data unambiguously demonstrates the existence of a smectic phase in a colloidal ellipsoidal system, which has been neither predicted by simulations nor previously found experimentally. In the smectic phase, the SAXS pattern exhibits a curious peak shape that resembles a *paper-clip* with highly anisotropic tails along the direction of the smectic periodicity. The presence of this unusual peak structure is rationalized by the modulation of the intensity in the form of a spherocylinder because of the correlation between the particles along different directions together with a layer undulation. In contrast, particles with a higher aspect ratio of ρ = 3.69 were found to self-assemble in only two different phases — *para-nematic* and *nematic*. While the theoretical phase diagrams for ellipsoids without external fields show the same sequence of phases and only small quantitative differences in the location of the corresponding phase boundaries within this range of axial ratios, this seems to change dramatically with the existence of an external field. In order to understand the origin of these observations, we have therefore combined our SAXS experiments with computer simulations, where we use two different geometrical models, namely, ellipsoids and spherocylinders, to investigate the effects of an external field and an increasing concentration on the phase behavior of these systems. The choice of these two geometrical models was motivated by the results of a detailed quantitative shape analysis of our two experimental model systems using transmission electron microscopy. Although the overall shape of the particles for ρ = 2.9 is ellipsoidal, a detailed inspection revealed that their shape also possesses some features similar to that of spherocylinders. As a result, the system exhibits a combination of all field-induced LC phases found for the pure ellipsoids and spherocylinders. On the contrary, particles with ρ = 3.69 possess a much closer resemblance to ellipsoids, which makes them exhibit a field-induced phase behavior similar to that found in simulations for ideal polydisperse ellipsoids. Our study indicates that small but systematic deviations of the actual particle shape from the ideal ellipsoidal shape combined with the unusual magnetic properties of hematite are at the origin of this hitherto not observed possibility to create an (oblate) smectic phase with prolate particles, demonstrating the importance of subtle effects of shape anisotropy in field-directed self-assembly.

**Figure 1:**
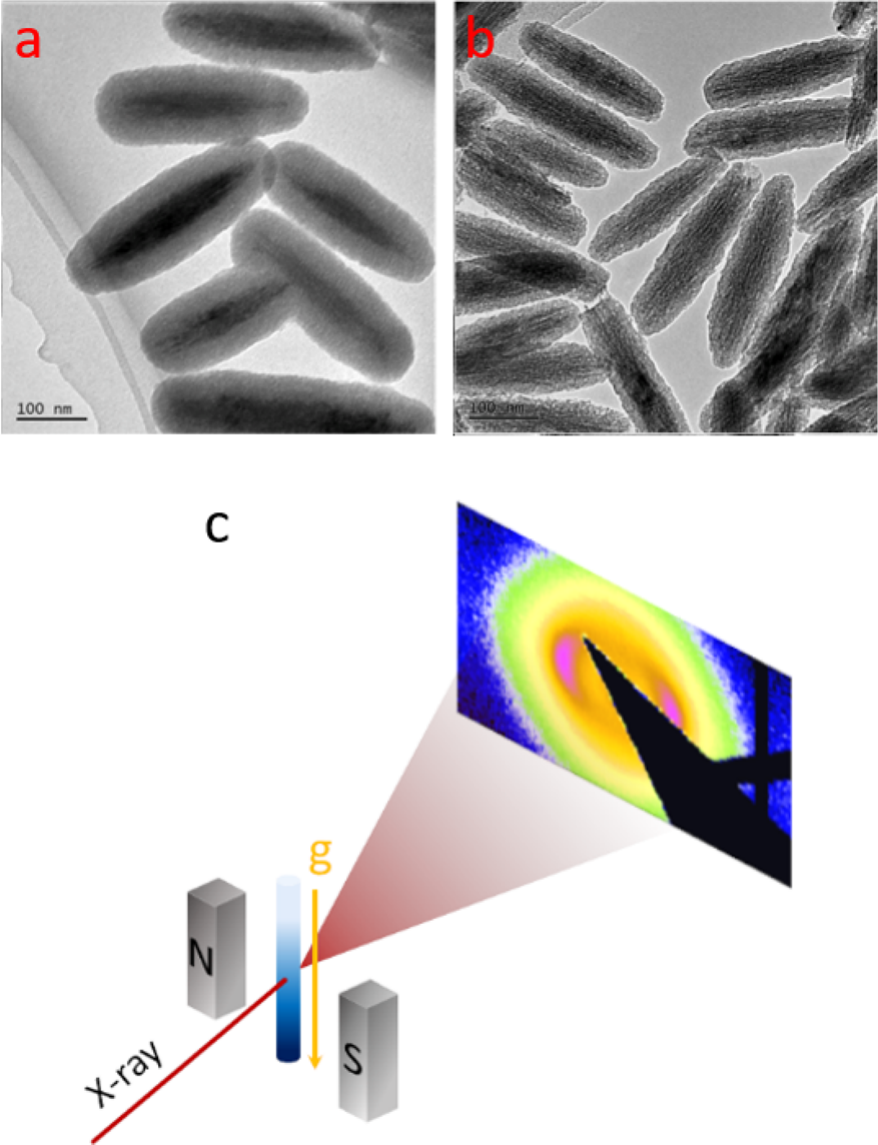
TEM images for ellipsoidal colloids for aspect ratios (a) ρ_1_ = 2.9 and (b) ρ_2_ = 3.69. (c) Experimental setup for SAXS measurement.

## Results and Discussion

### Sample *E*1 with Aspect Ratio ρ_1_ = 2.9

**Figure 2:**
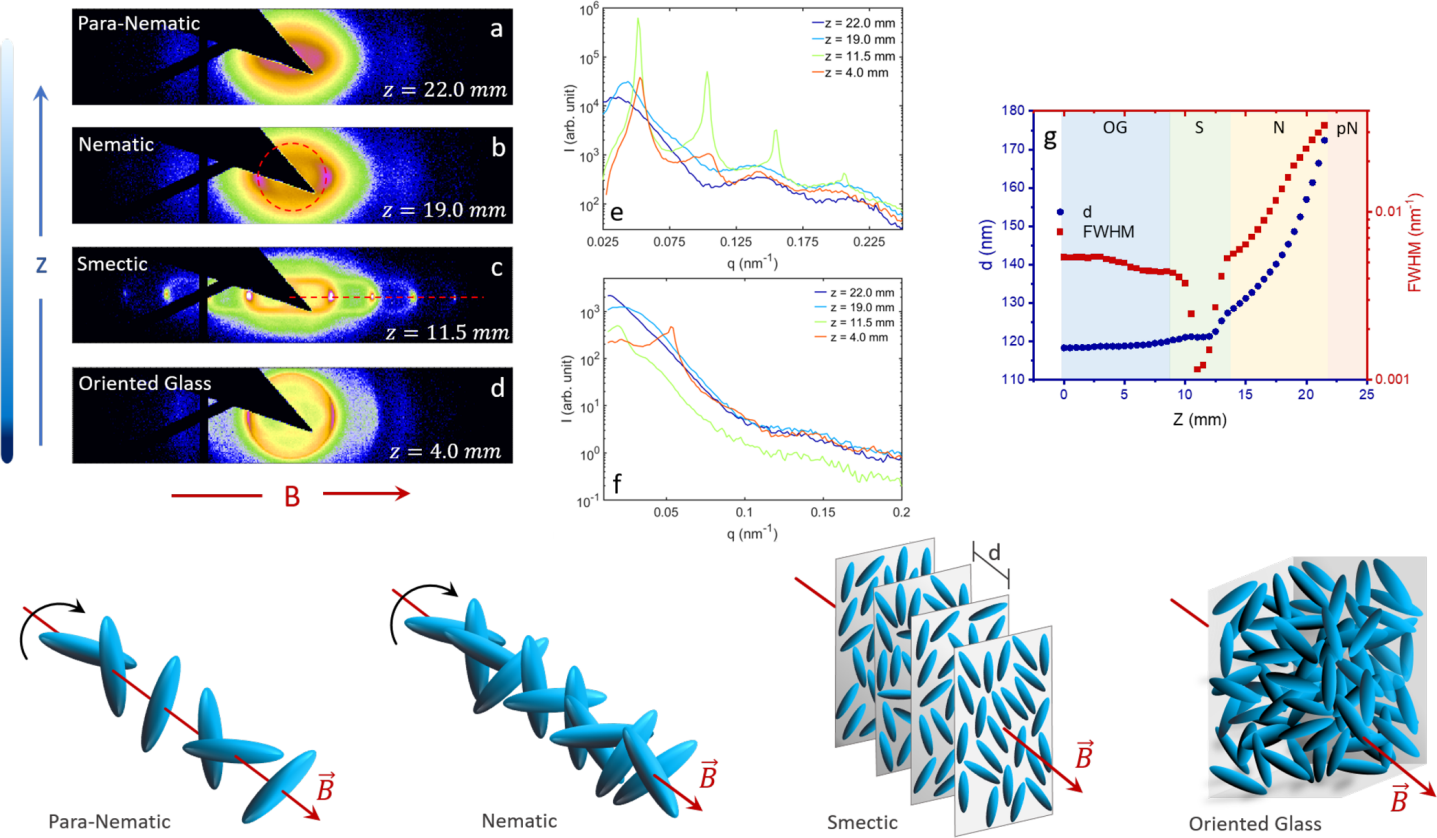
Typical 2D diffraction patterns for (a) para-nematic, (b) nematic, (c) smectic, and (d) oriented glass phases at different heights of the sedimented sample for ρ = 2.9. The variation of the scattered intensity as a function of scattering vector, *q*, for the aforementioned self-assembled phases along the direction of the external field (e) and perpendicular to it (f). (g) The variation of the nearest neighbor distance, *d*, and the FWHM of the nearest neighbor peaks as a function of height, *Z*, from the bottom of the capillary have been represented by the blue circles and the red squares, respectively. The bottom panel represents schematic of the aforementioned self-assembled phases.

The ellipsoidal system studied here exhibits a rich phase behavior involving para-nematic (pN) (the pN phase has been described before as a polarized fluid in ref (39)), nematic (N), smectic (S), and oriented glass (OG) phases or states as a function of height, *Z*, from the bottom of the capillary, *Z* = 0 mm being the extreme bottom. At the top of the sedimentation profile, the concentration is relatively low, resulting in weak interparticle correlations. As a result, the field-induced torque is mostly exerted onto single particles, resulting in an alignment that leads to the formation of a para-nematic phase (Figure 2a). A nematic phase extending over *Z* = 21.5 mm to *Z* = 14.0 mm followed by a smectic one from *Z* = 13.5 mm to *Z* = 9.5 mm is observed as one moves lower down in the capillary (Figure 2b,c). At the bottom of the sediment, due to the very high concentration, the particles form a kinetically arrested glass phase, which is observed between *Z* = 9.0 mm to *Z* = 0.0 mm. In the presence of an external field, the particles tend to align with their short axes parallel to the field direction. As a result, the glass phase develops an anisotropy (Figure 2d). The size of our X-ray beam (about 0.5 mm) was quite small compared with the spatial extension of the different phases in the capillary. The nematic phase found for colloidal rods, which align their long axis, is often referred to as a *prolate nematic*, N_+_, while plate-like colloids exhibit an *oblate nematic*, N_–_, where the short particle axes are aligned. It is interesting to note that despite the prolate shape of our ellipsoidal particles their orientational behavior in the presence of the external field closely follows that of oblate particles. This behavior can be rationalized when one considers the fact that the ellipsoids consist of a hematite core. As a result, their magnetic moments lie along the short axes of the particles. Under the influence of an external magnetic field these particles self-assemble with their short axes parallel to the field direction, thereby resembling oblate particles rather than rods. One can therefore refer to the field induced nematic, smectic, and para-nematic phases as N_–_, S_–_, and pN_–_ respectively, which is in strong contrast to the phases observed for colloidal rods.^41^

Figure 2e,f represent one-dimensional intensity profiles for different phases along and perpendicular to the direction of the field, respectively. At the very top of the sediment, in the pN phase, one can observe only the form factor, which is highly anisotropic; extending to larger *q* along the field direction and decaying much faster in a direction perpendicular to the field direction (Figure 2e,f (red)). This indicates that the particles are aligned with their short axes parallel to the field direction. At sufficiently high concentrations, positional correlations start to play an important role for the scattering in the direction parallel to the magnetic field. As a result, the static structure factor dominates the intensity profile, and we observe the appearance of additional well-developed maxima. The presence of very sharp smectic reflections up to fourth order along the direction of the field indicates a highly ordered structure (Figure 2e (cyan)). These strong positional and orientational correlations disappear again at the highest concentrations in the OG phase, where only a clear structure factor peak related to side-by-side correlations of the particles is observed parallel to the field direction (Figure 2e (blue)). In contrast, the scattering features are much less clearly developed in a direction normal to the field direction, and we only observe weak correlation peaks originating from the tip-to-tip configurations, that is, with a separation distance equal to the particle length, for the nematic, smectic and oriented glass phases (Figure 2f).

In a vertical scan, the nearest neighbor distance *d* (= 2π/*q*_max_) along the direction of the field increases as a function of the sample height *Z* (Figure 2g), reflecting the decrease in concentration with increasing *Z*. The full-width at half-maximum (FWHM) of the diffraction peak, which represents the inverse of the positional correlations, also changes as a function of *Z* (Figure 2g). For the oriented glass and the nematic phase, the FWHM is quite large, indicating the presence of liquid-like positional order. In case of the smectic phase, however, there is a sharp decrease in FWHM, demonstrating the existence of a one-dimensional crystalline order.

#### Characterization of the Nematic Phase

**Figure 3:**
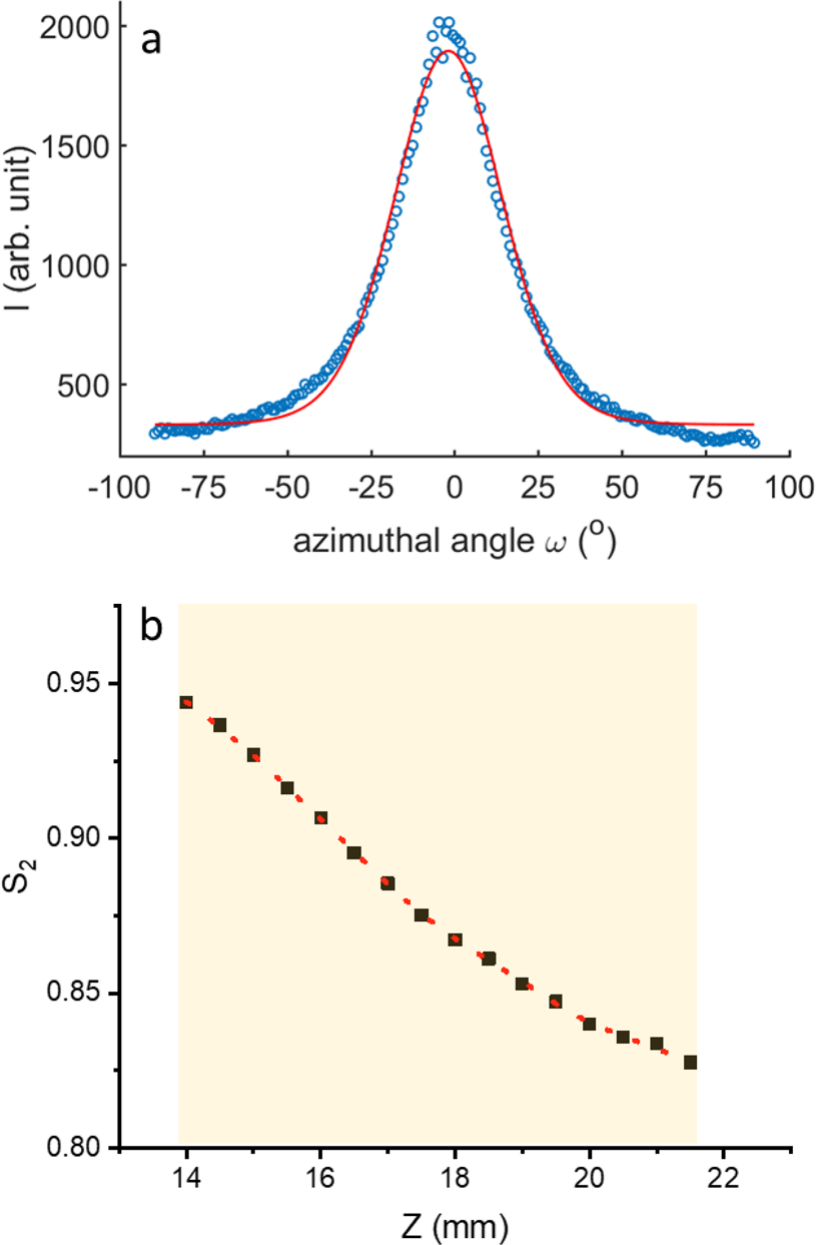
(a) The blue circles represent the azimuthal intensity profile along the red dashed line shown in Figure 2b and the corresponding fit using eq 1(red line). (b) The variation of the orientational order parameter *S*_2_ as a function of *Z* in the nematic phase. The red dashed line is a guide to the eye but not a fit.

The nematic phase is characterized by short-range positional order but long-range orientational order, which can be represented with the help of an orientational order parameter, *S*_2_. Following Purdy et al.,^42,43^ the azimuthal intensity distribution (along the red dashed line in Figure 2b) can be fitted with
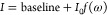
1where *I*_0_ is the normalizing factor, ω is the azimuthal angle, and *f*(ω) is the orientational distribution function
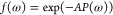
2where the parameter *A* determines the width of the distribution function and *P*(ω) is the Legendre polynomial
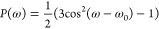
3with ω_0_ being the tilt angle. The orientational order parameter, *S*_2_, can now be obtained by
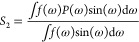
4Figure 3a shows a representative fit of the experimental data with the Purdy model, while Figure 3b represents the variation of *S*_2_ as a function of *Z*. The analysis reveals that one of the short axes of the ellipsoids are very well aligned throughout the nematic phase, resulting in an order parameter >0.8. An interesting observation is that the order parameter is larger at the bottom part, indicating that the sample is more ordered, which is expected because of the sedimentation-induced concentration gradient.

#### Characterization of the Smectic Phase

**Figure 4:**
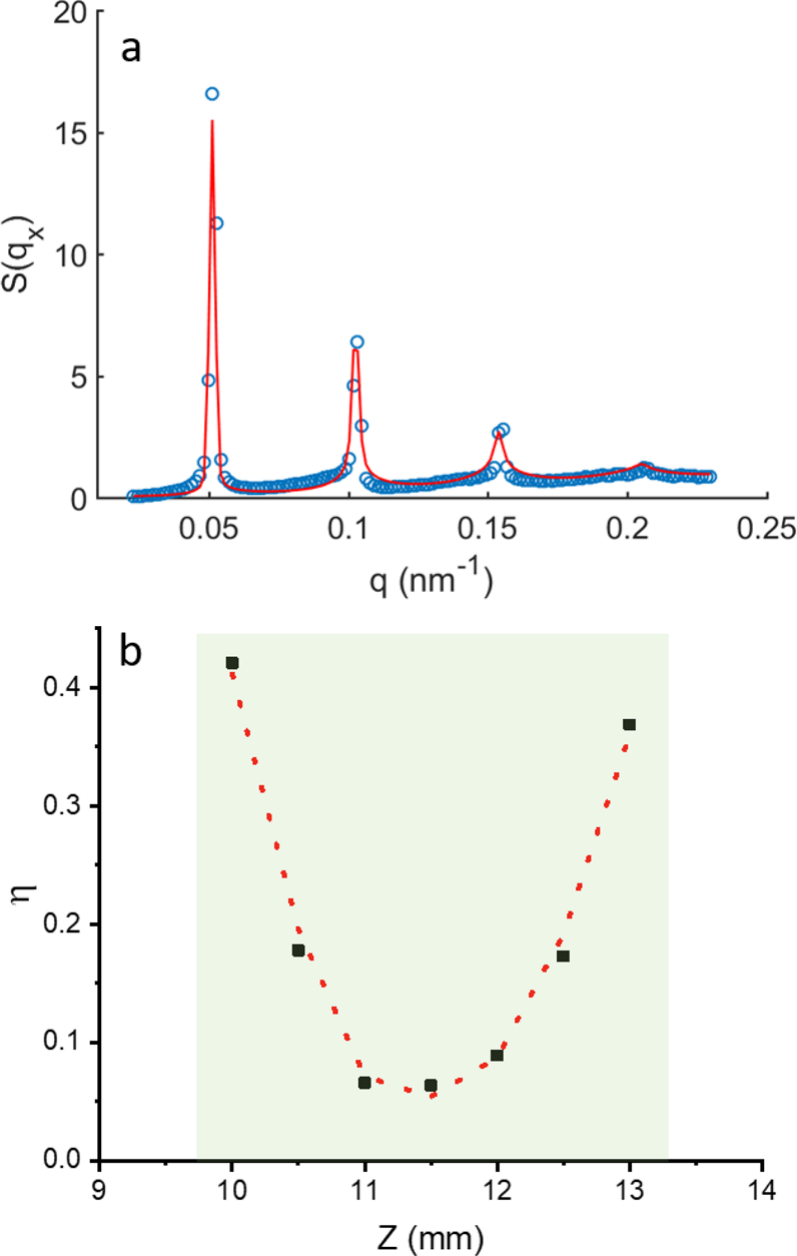
(a) Experimental structure factor along the field direction shown by red dashed line in Figure 2c (blue circles) along with the fit (red line) using the Nallet model described by eq 5 for the smectic phase at a height *Z* = 11.5 mm from the bottom of the capillary. (b) Variation of the Calliè parameter as a function of *Z* for the smectic phase. The red dashed line is a guide to the eye but not a fit.

The smectic phase is most commonly observed for amphiphilic systems where amphiphiles self-assemble in the form of bilayers, which then stack together to form a lamellar phase. We have characterized the smectic phase by borrowing the knowledge from the lamellar phase. The radially integrated structure factor *S*(*q*_*x*_) of the smectic phase can be obtained by dividing the recorded intensity, *I*, by the form factor (∝ *q*_*x*_^–2^). The resolution (*Δq*) limited *S*(*q*_*x*_) can now be described by the Nallet model,^44^
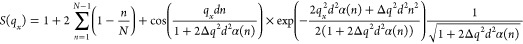
5with . Here, γ is Euler’s constant and η is the Caillé parameter, which is a measure of the fluctuations in the system; η = 0 means that there are no fluctuations, while in the presence of fluctuations, η increases and the higher-order Bragg peaks are smoothed out. Figure 4a shows a representative fit of the experimental data with the Nallet model, while Figure 4b represents the variation of the estimated η as a function of *Z*. One can observe that just above the glass phase, η is quite high with larger fluctuations in the system, followed by a sharp decrease in η with increasing *Z*, representing a highly ordered smectic phase. However, fluctuations and hence η again increase to higher values close to the nematic phase.

**Figure 5:**
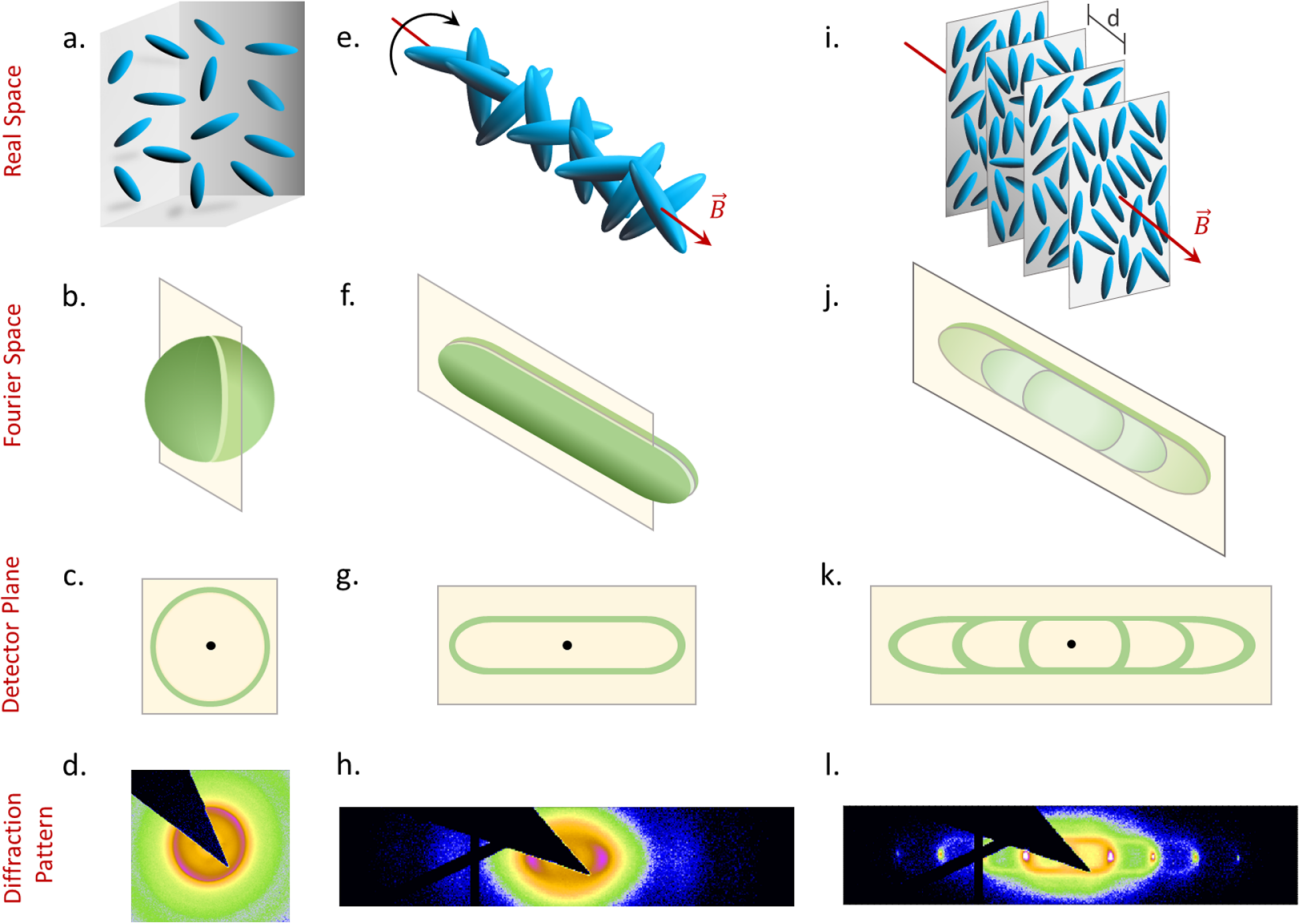
Top panel shows the (a) isotropic, field induced (e) nematic and (i) smectic phase for our colloidal prolate particles in real space. The second panel from the top shows the 3D Fourier space representation of the structure factor variation corresponding to an (b) isotropic, (f) nematic, and (j) smectic phase. In the presence of the external field, since the particles get aligned with their short axes being parallel to the field direction for the nematic and smectic phases, the corresponding 3D Fourier space structure factor vary in the form of a cylinder. For the smectic phase, once the layers start to get correlated, sharp Bragg spots start to appear on the axis of this cylindrical structure factor. The light yellow planes indicate the detector planes which pass through the Fourier structures. The third panel from the top indicates the expected diffraction patterns on the detector plane for a (c) isotropic, (g) nematic, and (k) smectic phase. The sharp Bragg spots become arcs because of polycrystallinity. The bottom panel shows the experimental diffraction for the (d) isotropic, (h) nematic, and (l) smectic phase.

Further, a careful inspection of the diffraction pattern of the nematic and smectic phases reveals an unusual but interesting feature resembling *paper-clip* (Figure 2b,c) shapes. This peculiar shape of the diffraction pattern is more prominent in the smectic phase, where one can clearly notice diffuse scattering lines parallel to the direction of the smectic periodicity. In the absence of the external magnetic field, particles are randomly oriented in all possible directions (Figure 5a). As a result, in Fourier space, the structure factor is expected to be modulated in the form of spherical shells as one can see in Figure 5b, which in turn produces a circle on the detector plane (shown by the yellow planes in Figure 5b,c). Since a sphere is a three dimensionally symmetrical object, it does not matter at which angle the detector plane is passing through it to get a circular/isotropic diffraction pattern (Figure 5d). In the presence of the external field, the particles tend to align with their short axes parallel to the field direction (Figure 5e). In turn, one of the rotational degrees of freedom freezes out, resulting in anisotropy in the Fourier space structure. Along the magnetic field, the correlation distance is governed by the smaller particle dimension, while in the orthogonal directions it is mainly dominated by their length. In the intermediate directions between these two, the gradual change of the typical correlation distance leads to this peculiar shape of the structure factor that resembles a spherocylinder (SC) as shown in Figure 5f. Depending on the direction at which the detector plane intersects the Fourier space, one would therefore expect to observe either a circle, ellipse, or a two-dimensional projection of a SC on the detector plane. For our experimental geometry, the detector plane passes parallel to the long axis of the three-dimensional spherocylinder generated in Fourier space, and one would thus expect to see a structure factor modulated in the shape shown in Figure 5g. This expected diffraction pattern matches exactly with what we observe in the experiment for the nematic phase (Figure 5h).

With a further increase in concentration (i.e., moving down in the capillary), the particles get confined in 2D planes with their long axes lying on the planes (Figure 5i). These planes are normal to the field direction, and within each plane, particle ordering is *liquid*-like. Beyond a certain concentration, correlation starts to build up between these planes, resulting in a smectic phase. In an ideal smectic structure, one would expect a sequence of sharp smectic reflections together with a broad intralayer scattering peak that is mostly broadened in a direction perpendicular to the layering direction. In contrast, what we observe is a paper-clip-like diffraction pattern. This peculiar shape suggests the presence of strong nematic-like fluctuations such as undulation of the smectic layers and correlations between the particles of different layers. While the correlations between the particles (that belong to different layers) along different directions lead to the horizontal diffused scattering line along the smectic periodicity, the layer undulations will lead to the appearance of the tails for higher-order Bragg peaks, which explains the multiple paper-clip-like features as highlighted in Sec. I of SI. The corresponding Fourier space structure is shown in Figure 5j. The detector plane being parallel to the field direction and perpendicular to the X-ray beam (Figure 1c) passes through the SC along its long axis to produce a diffraction pattern in the form of a paper-clip (Figure 5k) as one can observe in our experimental scattering pattern (Figure 5l).

### Sample *E*2 with Aspect Ratio ρ_1_ = 3.69

**Figure 6:**
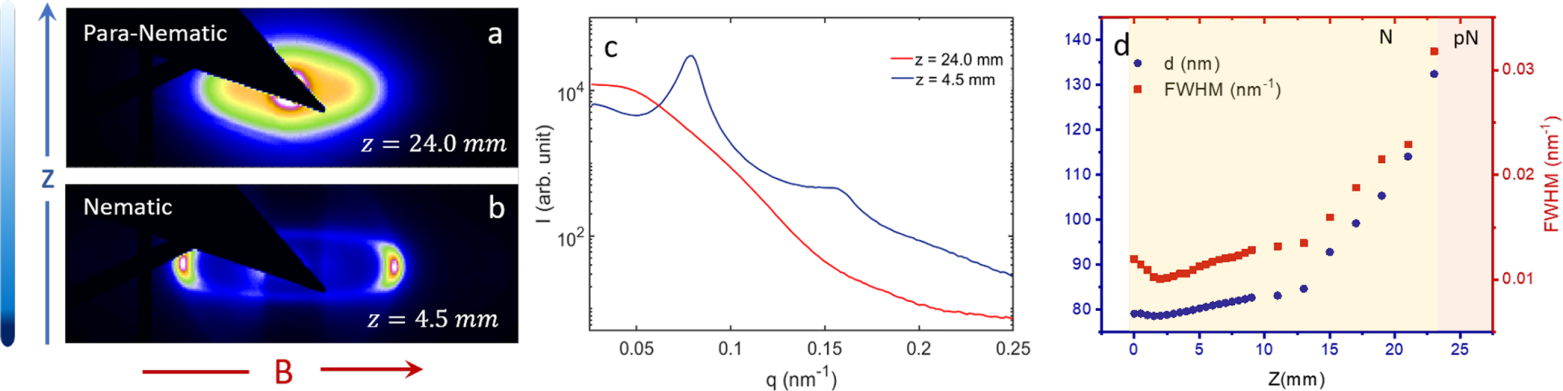
Typical 2D diffraction patterns for (a) para-nematic and (b) nematic phases at different heights of the sedimented sample for ρ = 3.69. (c) The variation of the scattered intensity as a function of scattering vector, *q*, for the aforementioned self-assembled phases along the direction of the external field. (d) The variation of the nearest neighbor distance, *d*, and the FWHM of the nearest neighbor peaks as a function of height, *Z*, from the bottom of the capillary have been represented by the blue circles and the red squares, respectively.

In contrast to the particles with lower aspect ratio, the ones with higher aspect ratio show only two different phases in the presence of the external field. At the top of the capillaries, a para-nematic phase is observed, followed by a nematic phase at the bottom (Figure 6a,b). In this case, the diffuse scattering due to the formation of the spherocylindrical structure factor is much more prominent in the nematic phase itself (Figure 6b). Figure 6c represents the one-dimensional intensity profile for the nematic and para-nematic phase along the direction of the field. The nearest neighbor distance, *d*, along the direction of the field increases with *Z* in a similar way as that of sample *E*1 (Figure 6d). In contrast, FWHM is increasing monotonically since sample *E*2 does not show any smectic phase (Figure 6d). While for our range of axial ratios, the theoretical phase diagrams for ellipsoids without external fields show the same sequence of phases and only small quantitative differences in the location of the corresponding phase boundaries,^32−34^ the existence of an external magnetic field results in dramatic changes, most notably the appearance of a hitherto unknown additional smectic phase for the smaller axial ratio. To understand the origin of this surprising field-induced additional smectic phase, we have performed systematic Monte Carlo (MC) computer simulations that are discussed in the following section.

### Computer Simulations

Following earlier studies^39^ of similar hematite-silica core–shell particles, we initially performed MC simulations using a model of polydisperse hard ellipsoids (HE). Polydispersity was included through a procedure where the length *L* and the diameter *D* of each particle were randomly drawn from a Gaussian distribution as follows:
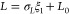
6
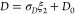
7where ξ_*j*_ with *j* = 1,2 is a zero-mean unit-variance Gaussian, *L*_0_ = 316 nm, σ_*L*_ = 26.3 nm, *D*_0_ = 108 nm and σ_*D*_ = 6.98 nm. The average aspect ratio of the simulated HEs is equal to 2.9, that is, close to the one of hematite-silica particles with the smaller aspect ratio investigated experimentally. In the following discussion, lengths will be expressed in units of 100 nm. We simulated *N* = 1125 particles in an orthorhombic box with periodic boundary conditions. In order to compare with the experimental system, we have included an external field with a tunable strength that aligns the HEs with their long axis perpendicular to the field direction. Its action is implemented through the following external potential acting on each particle *i*
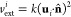
8where **n̂** is a unit vector parallel to the field, **u**_*i*_ is a unit vector parallel to the symmetry axis of the particle, and *k* is a parameter by which one can set the strength of the alignment. In our simulation, we used *k* = 10^4^*k*_B_*T* since in the real system the magnetic field induces a rather strong alignment of hematite-silica core–shell particles. We observe that hematite-silica core–shell particles are charged, but at high volume fractions, the resulting repulsive interaction between them can be accounted for by just considering a larger effective particle volume. Hence, we expect that electrostatic repulsion between particles induces a shift of phase boundaries to smaller packing fractions without altering qualitatively the whole phase behavior.

**Figure 7:**
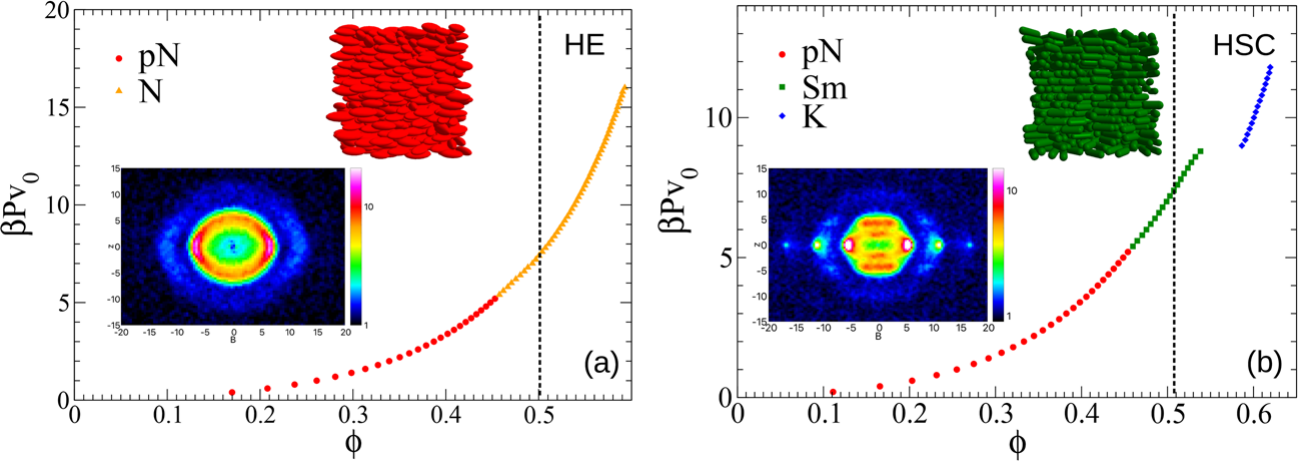
Equation of states of (a) hard ellipsoids and (b) hard spherocylinders from Monte Carlo simulations for ρ = 2.9. β, *P*, and *v*_0_ represent 1/*kT*, pressure and volume of a single particle, respectively. Insets show the static structure factors and snapshots for ϕ ≈ 0.50.

To build the equation of state (EOS) for these polydisperse HEs in the presence of the magnetic field, we performed MC simulations in the isobaric ensemble (NPT), where the three dimensions of the orthorhombic box were allowed to change independently in order to ease the formation of mesophases. Figure 7a summarizes the results obtained for the EOS of HEs. Similarly to what we have observed experimentally for hematite-silica particles with ρ = 3.72 (Figure 6) and in previous studies,^39^ the polydisperse HEs with ρ = 2.9 exhibit two phases throughout all pressures considered in the simulations: an oblate para-nematic (pN_–_) phase (for ϕ_0_ ≲ 0.45), where the symmetry axis of the HEs is aligned perpendicularly to the external field **B**, and an oblate nematic phase (N_–_) where HEs self-align also along a direction perpendicular to **B** (see Sec. II of SI for more information). It is interesting to note that the transition between these two phases is associated with a small variation of the volume fraction ϕ, thus suggesting that this transition is either weakly first order or second order.

Figure 7a also shows a characteristic snapshot and the corresponding static structure factor at a volume fraction of ϕ ≈ 0.5. The snapshot of HEs is a prototypical example of a nematic phase, whereas no lamellar ordering is present. The structure factor similarly reflects the absence of any layering in the system, and it can be straightforwardly compared with the experimental one shown in Figure 2b. Here we note that the structure factor that we calculated from the simulations includes the form factor and thus represents an effective structure factor as measured by SAXS and moreover corresponds to **q**-values lying onto the plane identified by the external field (B, i.e. *y*-axis) and a direction perpendicular to it (*z*-axis), so that a direct comparison with experiments can be made (see Sec. II of SI for more details). Our MC simulations are thus not capable to reproduce the occurrence of a smectic phase but instead reveal the same qualitative phase behavior as expected for HEs with larger aspect ratio, according to numerical studies reported in ref (39). The qualitatively different phase behavior for the two aspect ratios and the existence of an additional field-induced smectic phase observed in our experiments for particles with an aspect ratio of ρ = 2.9 is thus not in agreement with a model of polydisperse hard ellipsoids.

A subsequent close inspection of the actual particle images using TEM (Figure 2a,b) reveals that the two particle systems differ not only in size and aspect ratio, but that they also show subtle but systematic differences in their geometrical shape. Hematite-silica core–shell particles of aspect ratio ρ = 3.69 (see 2b and SI) closely resemble hard ellipsoids (HEs). In contrast, while the particles with the smaller aspect ratio ρ = 2.9 are overall still best described by a model of an ellipsoid, they possess a hybrid shape. Their midsection contains a considerably more cylinder-like (i.e., flatter) portion that resembles a hard spherocylinder (HSCs), while their ends are best described by a uniaxial hard ellipsoid model (HEs) as shown in Figure 2a. These qualitative differences are further described using a more quantitative image analysis approach described in detail in SI. In order to investigate the influence of shape more systematically, we have thus also performed MC simulations using a model of polydisperse hard spherocylinders (HSC), which resemble the straight sections present in our particles with ρ = 2.9 more closely (see Sec. IV in the SI). Polydispersity was implemented again as described above for the HEs. The results from these additional simulations are summarized in Figures 7b, where we show the EOS as well as a characteristic snapshot and the corresponding static structure factor at a volume fraction of ϕ ≈ 0.5. One can observe that the experimentally obtained 2D intensity profiles and the simulated static structure factors corresponding to nematic and smectic phases are indeed closely resembling each other (see Figure S19 in SI for a direct comparison).

In the explored range of pressures, the phase behavior of HSCs in the presence of an external field is richer when compared to HE, and we now find three phases: an oblate para-nematic (pN_–_) phase, analogous to the one observed for HEs; an oblate smectic A phase (SmA_–_), where the layers are perpendicular to the field; and a uniaxial columnar crystal phase (K). For the HSCs, we thus indeed observe the emergence of a lamellar phase for 0.45 ≲ ϕ ≲ 0.55 in agreement with the experimental results for hematite-silica particles with ρ = 2.9 (see also Sec. II of SI). Interestingly, the transition from the pN_–_ to the SmA_–_ phase (as the pN_–_-N_–_ one) is not associated with a significant variation of ϕ, so that it can be either very weakly first-order or second-order. Differently, the transition from the SmA_–_ phase to the crystal phase is markedly second-order as expected. It is also important to point out that the SmA_–_ phase observed in the simulations is purely a field-induced effect since it disappears once the external field is switched off (see Sec. III of the SI).

The formation of a smectic phase is further illustrated with the snapshot of HSCs shown in the inset of Figure 7b, where a clear lamellar ordering is apparent that is also reflected in the Bragg peaks present in the structure factor. The distance between Bragg peaks is equal to 2π/*d* with *d* ≈ 1 *r.u*., i.e. about *D*_0_, consistent with the experimental structure factor shown in Figure 2c for the smectic phase of hematite-silica particles. Our results clearly demonstrate that the cylinder-like midsection of HSCs favors the emergence of a lamellar phase, even if some amount of polydispersity is present in the system.

While the HSC simulations are capable of reproducing the occurrence of a field-induced smectic phase, a comparison between the experimental observations (Figure 2) and the simulation results (Figure 7b) also reveals clear differences. Whereas the hematite-silica particles with ρ_1_ = 2.9 undergo a transition to a nematic phase, this phase is absent for HSCs, also in agreement with the phase diagram of monodisperse HSCs. However, such a nematic phase is present for HE, whose phase diagram mimics the one observed for the hematite-silica particles with ρ = 3.69. This clearly indicates the importance of the actual shape and of small but systematic local deviations from the ideal geometrical models. While these hematite-silica particles overall resemble ellipsoids, and have previously been used successfully in a number of studies as experimental model systems for ellipsoidal colloids, field-driven self-assembly at higher volume fractions is obviously much more sensitive. Here the hybrid nature of the experimental particles becomes important, and the subtle shift from a particle that is locally better described by a spherocylinder (ρ_1_ = 2.9) to one where the ellipsoidal geometry dominates (ρ = 3.69) is decisive for the resulting field-induced phase behavior (see also SI for more information on the quantitative image analysis for both particles).

On the basis of our findings from simulations and experiments, we believe that the unexpected sequence of field-directed structures is a fortuitous consequence of the two-step synthesis process used, where the overall shape and dimensions of the particles are first established through the synthesis of the hematite spindle core, and the final aspect ratio is then selected through a coating with a silica layer of variable thickness. In addition to the change in the axial ratio, the silica coating however also induces small but systematic local deviations from the overall ellipsoidal shape, favoring straighter sections that resemble a cylinder rather than an ellipsoid for larger shell thicknesses. While for the particles with the thinner silica shell and thus the larger aspect ratio, these local shape variations are not sufficient to influence the phase behavior in the presence of the magnetic field, this changes dramatically for the thicker shell and smaller aspect ratio. Here the hybrid nature of the particle shape results in a more complex phase behavior that is also hybrid in nature. At lower volume fractions, the ellipsoid-like ends of the particles appear to dominate, and we observe the formation of a nematic phase that is characteristic for ellipsoids. At higher volume fractions, however, the cylinder-like middle section now allows for the formation of a field-induced smectic phase that is characteristic for hard spherocylinders.

## Conclusions

In this article, we report and discuss the polymorphism exhibited by hematite-silica core–shell prolate particles undergoing field-directed self-assembly. When comparing the results obtained for two different aspect ratios, we surprisingly found not only quantitative shifts of the different phase boundaries but also a smectic phase that has not been reported previously for prolate ellipsoidal colloids. On the basis of a comparison of our experimental findings with MC simulations of simple anisotropic models with comparable axial ratios and polydispersity, we are led to conclude that the surprising experimental observations cannot be linked to the influence of axial ratio and external field only. Instead, we believe that our results indicate the importance of subtle but systematic imperfections in the particle shape for the behavior of anisotropic particle systems in field-directed self-assembly.

On a more technical level, a detailed analysis of the SAXS data has provided us with an estimation of key structural quantities such as the orientational order parameter, *S*_2_, and the fluctuation parameter, η, for the nematic and smectic phases, respectively. We have found that *S*_2_ has very high values (>0.8) throughout the whole nematic phase, indicating a highly ordered nematic phase. The ordering of the smectic phase is less at the phase boundaries and increasing as one is moving away from the same, as suggested by the variation of η. In addition, because of the freezing of one of the rotational degrees of freedom, an unusual diffuse scattering pattern in the form of a spherocylinder is observed. We believe that the variation of the structure factor in this particular form is a quite general diffraction feature as it has been observed for other anisotropic colloids where one out of three rotational degrees of freedom is frozen.^24^

Overall, our study has clearly indicated that it is not enough to consider global shape characteristics (e.g., axial ratios) when striving to understand and exploit (field-directed) self-assembly in order to fabricate ordered phases at high packing fractions. Experimental properties such as rotational and translational diffusion coefficients or structure factors of real particle dispersions such as the ones used in this study are indeed well described by models such as hard ellipsoids.^38−40,45^ However, this is no longer the case when we try to understand and predict their phase behavior in field-directed self-assembly. Our findings should serve not only as a warning when comparing experimental results obtained with “real” particles that always carry small shape imperfections with computer simulations using generic geometrical models but also, more importantly, as an indication that subtle variations of the resulting shape may lead to a much greater diversity in the accessible range of ordered phases that can be created through field-directed self-assembly.

## Methods and Experimental

### Synthesis and Characterization Methods

Silica/hematite core/shell ellipsoidal particles of two different aspect ratios were synthesized. Hematite ellipsoids were initially synthesized in water following the approach described by Ocana et al.^46^ They were then coated with silica layer(s) in ethanol using the method described by Graf et al.^47^ The aspect ratio of the particles was controlled by tuning the thickness of the silica shell. Particles were purified by repeated centrifugation/redispersion cycles in water and were kept in water as a stock dispersion. Details of the synthesis and characterization of similar particles can be found elsewhere.^38^

A transmission electron microscope (TEM) (TEM-CM100 microscope from Philips operating at 100 keV) was used to characterize both the size and shape of the particles. Particle size distributions were calculated by measuring at least 100 particles from TEM micrographs using the software ImageJ. For the batch of particles that we have named *E*1, we find the long and short axes to be *L*_1_ = 316 ± 26.3 nm and *D*_1_ = 108 ± 7 nm respectively, leading to an aspect ratio of ρ_1_ = 2.9, while for another batch, named *E*2, *L*_2_ = 266 ± 19 nm and *D*_2_ = 72 ± 6 nm, corresponding to ρ_2_ = 3.69. Figure 1a,b shows representative TEM images for the ellipsoids.

### Microradian Small-Angle X-ray Scattering

For SAXS measurements, dispersions of sample *E*1 at 55 wt % and sample *E*2 at 50 wt % were placed in round capillaries with an internal diameter of 1 mm (Mark tubes) that were flame-sealed and stored vertically and left undisturbed for 6 months to allow for particle sedimentation. Gravity-induced sedimentation in the colloidal dispersions results in concentration gradients that leads to the formation of different self-assembled structures. In order to investigate the self-assembled structures, the sedimentation profiles were scanned over the full length of the capillary along the vertical directions with *Z* = 0 being set at the bottom of the capillaries. SAXS measurements were performed at BM26B beamline at ESRF, Grenoble, where a μrad-SAXS setup exploiting compound refractive lenses^48,49^ was employed. The 13 kev X-ray beam was focused on a CCD X-ray detector which was placed at a distance of 7.45 m from the sample. The data have been recorded with a PILATUS 1 M detector with pixel size of 172 × 172 μm. The detector was protected from the direct X-ray beam using a wedge-shaped beam-stop that shades the detector. The capillaries were oriented vertically with their long axis (100 mm) parallel to the gravitational field. All the measurements were carried out in the presence of an external magnetic field (500 mT) which was applied after the sedimentation using a permanent magnet. The direction of the magnetic field, X-ray beam and the gravity are perpendicular to each other as shown in Figure 1c.

### Computational Methods

All the results from simulations were obtained by performing up to 4 × 10^7^ MC steps and by discarding the initial equilibration stage. The initial configurations, which we used to build the EOS, were obtained by equilibrating the system at low volume fractions to have an isotropic sample. To calculate the static structure factor we carried out NTV MC simulations starting from an initial configuration taken from NPT simulations for a *P* such that ϕ ≈ 0.50. Aiming at a more direct comparison with experimental results we calculated the static structure factors by replacing each particle (HSC or HE) with a random set of scattering points of fixed density ρ_*m*_. We found that results do not change significantly by using values of ρ_*m*_ greater than about 7 × 10^–5^ nm^–3^. Concerning the algorithms which we used for checking the overlap of HSCs and HEs, we employed the one proposed by Vega and Lago^50^ for HSCs and the one proposed by Perram and Wertheim.^51^ Further details on computer simulations can be found in the Supporting Information.

## Supporting Information Available

Real and Fourier space structures of smectic phase; detailed simulation results on static structure factor, pair distribution function, smectic and nematic order parameters in the presence and absence of external magnetic field; detailed shape analysis of prolate particles using TEM images; comparison of the experimentally obtained intensity profiles and the simulated static structure factors for the nematic and smectic phases (PDF)
