# Supplementary material for: Shape Matters in Magnetic-Field-Assisted Assembly of Prolate Colloids
Source: ACS Nano. 2022 Feb 9;16(2):2558–68. doi: 10.1021/acsnano.1c09208 (PMC8867904; doi:10.1021/acsnano.1c09208)
Supplement: Supplementary file 1 — nn1c09208_si_001.pdf [file nn1c09208_si_001.pdf]

Shape Matters in Magnetic-Field Assisted Assembly of Prolate Colloids

Antara Pal,[1,a)] Carlo Andrea De Filippo,[2] Thiago Ito,[1] Md. Arif Kamal,[3] Andrei V. Petukhov,[4,5] Cristiano De Michele,[6] and Peter Schurtenberger[1,7,b)]

[1)] *Division of Physical Chemistry, Department of Chemistry, Lund University, Lund, Sweden*

[2)] *Dipartimento di Scienze, Università degli Studi Roma Tre, Via della Vasca Navale 84, Roma, Italy*

[3)] *Centre Interdisciplinaire de Nanoscience de Marseille (CINaM), CNRS, Aix Marseille University, Marseille, France*[c)]

[4)] *Van't Hoff Laboratory for Physical and Colloid Chemistry, Utrecht University, Utrecht, The Netherlands*

[5)] *Laboratory of Physical Chemistry, Eindhoven University of Technology, Eindhoven, The Netherlands*

[6)] *Department of Physics, Università di Roma La Sapienza, I-00186 Roma, Rome, Italy*

[7)] *Lund Institute of advanced Neutron and X-ray Science LINXS, Lund University, Lund, Sweden*

[a)]Electronic mail: antara.pal@fkem1.lu.se

[b)]Electronic mail: peter.schurtenberger@fkem1.lu.se

[c)]Current Address: Division of Physical Chemistry, Department of Chemistry, Lund University, Lund, Sweden

I. REAL AND FOURIER SPACE STRUCTURES OF SMECTIC PHASE

The real space structure of a classical smectic phase consisting of rod-like colloids is schematically shown in Fig. S1(a). In this case the particles align along their long axes. As a result, the smectic layers form in a direction parallel to the length of the rods. The corresponding Fourier space image or the expected x-ray diffraction pattern of the aforementioned smectic phase is shown in Fig. S1(b)[1,2]. However, the smectic phase formed by ellipsoidal particles which in the presence of an external field align with their short axes parallel to the external field, has the appearance as shown in Fig.S1(c). The double headed red arrows indicate the spacings along the smectic layers. Correlations between particles which belong to different layers (as indicated by green lines) results in the formation of a diffused scattering line as shown in Fig.S1(d) in green. Although our schematic gives an impression that the long axes are also aligned, but this is not the case in general. As mentioned before the particles align with their short axes along the field direction and their long axes are free to rotate about this direction (Fig. 2). For the sake of clarity in illustration, we have chosen to highlight only one such possible conformation out the ensemble of all possible rotational conformations. Further it is important to note that the smectic layers in this case are not rigid but can fluctuate, Fig.S1(e), resulting thereby in an elongation of the smectic peak in vertical direction as indicated by dark yellow in Fig.S1(f).

II. CHARACTERIZATION OF THE PHASE BEHAVIOR BY SIMULATION

A. Static Structure factor

A way to characterize the smectic phase obtained from the simulations is provided by the calculation of the static structure factor[3]:

$$S(\vec{q}) = \left\langle \frac{1}{N} \rho_{\vec{q}} \rho_{-\vec{q}} \right\rangle \tag{1}$$

where N is the number of scattering points, $\langle \ldots \rangle$ is an average over independent configurations and $\rho_{\vec{q}}$ is the Fourier transform of the microscopic density:

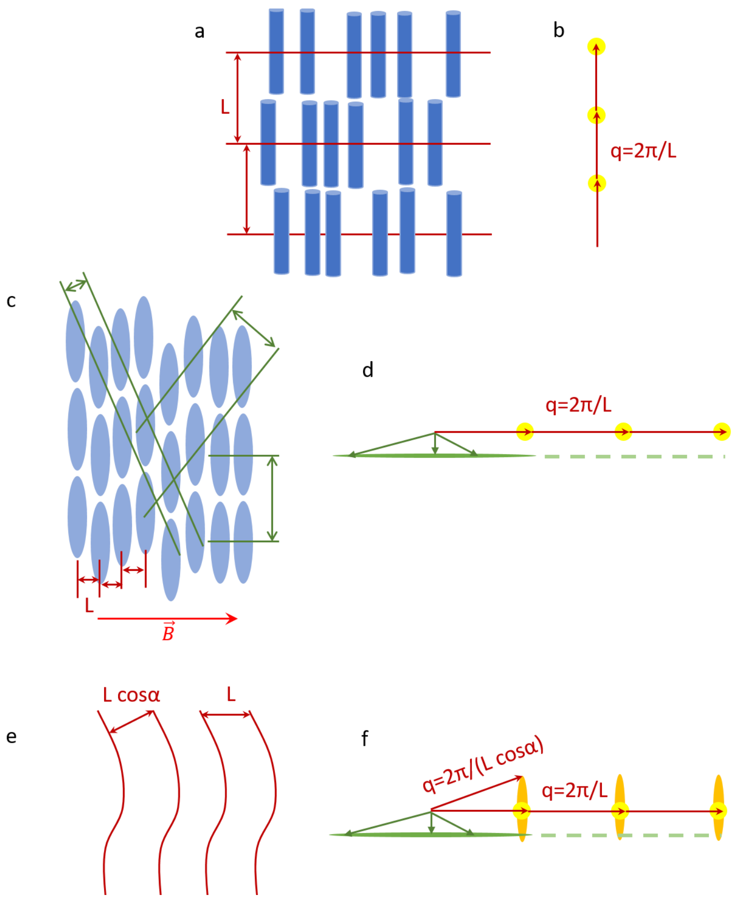

FIG. S1. (a) Real space image of a classical smectic phase formed by colloidal rods and (b) expected x-ray diffraction pattern for it. (c) Real space image of oblate smectic phase formed by ellipsoids and (d) expected diffraction x-ray diffraction pattern for it. (e) Smectic phase with layer fluctuation and (d) the corresponding change in the diffraction pattern.

$$\rho_{\vec{q}} = \sum_{i=1}^{N} \exp(-i\,\vec{q}\cdot\vec{r}_i) \tag{2}$$

Monte Carlo simulation in the NVT ensemble for both hard ellipsoids (HEs) and hard spherocylinders (HSCs) have been carried-out starting from a configuration at $\phi = 0.50$. From these simulations we obtained many independent configurations for the centers of mass of both HEs and HSCs. In these configurations we replaced each particle with a set of scattering points randomly placed inside it and of fixed number density, as shown in Figs. S2 and S3. Finally, by using these random sets of points in Eq. 1 we calculated the static structure factor $S(q)$ onto the plane yz parallel to the magnetic field (i.e. we calculated $S(0, q_y, q_z)$), where, here and in the following, the y-axis is assumed to be parallel to the

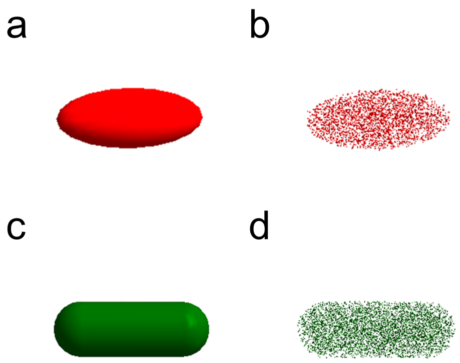

FIG. S2. 3D representations of a ellipsoid (a) and a spherocylinder (c) and their corresponding random sets of points, shown in panels (b) and (d), respectively.

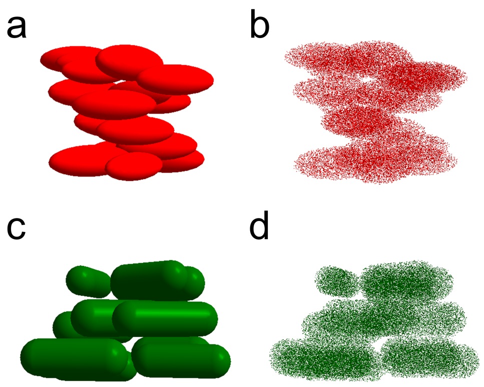

FIG. S3. A set of HEs (a) and HSCs (c) represented as 3D solids and as random sets of points shown in panels (b) and (d), respectively.

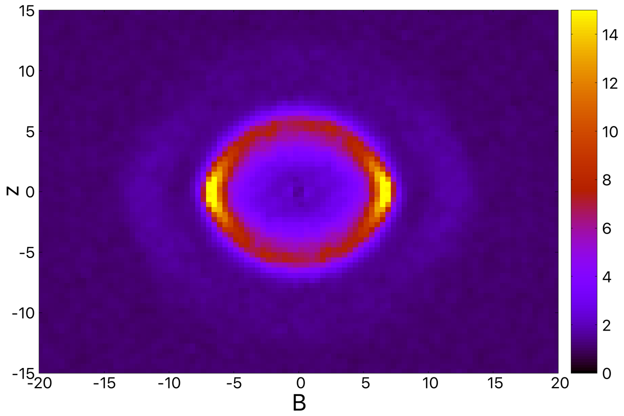

FIG. S4. Structure factor $S(0, q_y, q_z)$ of a system of HEs with a magnetic field along the y (B) axis at $\phi = 0.50$.

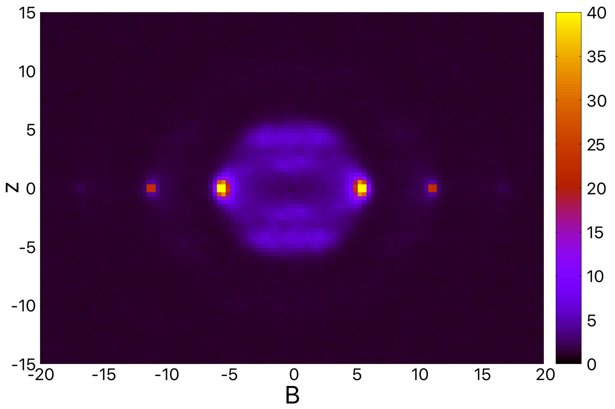

FIG. S5. Structure factor $S(0, q_y, q_z)$ of a system of HSCs with a magnetic field along the y axis at $\phi = 0.50$.

external magnetic field. The results are shown in Fig. S4 for HEs and in Fig. S5 for HSCs.

B. Pair distribution function

The phase behavior can be also characterized by calculating the three-dimensional pair distribution function $g(\vec{r})$, i.e.:

$$g(\vec{r}) = \frac{1}{\rho N} \left\langle \sum_{i=1}^{N} \sum_{j \neq i} \delta \left(\vec{r} - (\vec{r}_i - \vec{r}_j) \right) \right\rangle \tag{3}$$

where $\delta(x)$ is the Dirac delta function and $\langle \ldots \rangle$ is an average over independent configurations. We calculated the $g(\vec{r})$ onto the planes yz and xz, which are parallel and perpendicular to the magnetic field, respectively. The results for the HSCs are shown in Fig. S6 and Fig. S7 from which the smectic ordering is rather apparent. Differently, the radial distribution function of HEs does not show any layering for all state points studies. A typical example of $g(r)$ for HEs is shown in Fig. S8 (yz-plane) and Fig. S9 (xz-plane). The anisotropy and structuring shown in Fig. S8 reflects the alignment of HEs due to the magnetic field. Differently, the anisotropy of the radial distribution function calculated onto a plane perpendicular to the magnetic field, which is shown in Fig. S9, is due to orientational ordering of HEs, which builds up at volume fractions higher than ≈ 0.45. More details on this are provided below (see subsection I-D entitled "Smectic and nematic order parameters").

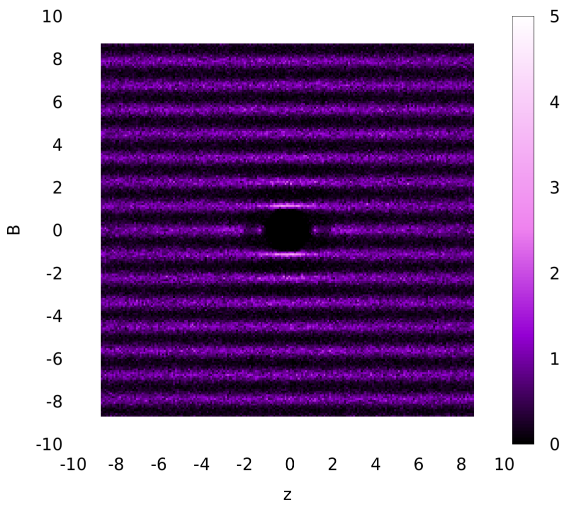

FIG. S6. Pair distribution function for a system of HSCs $\phi = 0.50$ with a magnetic field $\vec{B}$ directed along y axis.

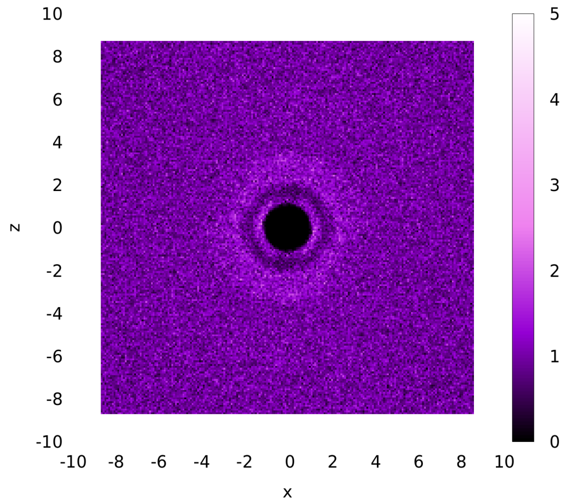

FIG. S7. Pair distribution function for a system of HSCs $\phi = 0.50$ with a magnetic field $\vec{B}$ directed along the y-axis.

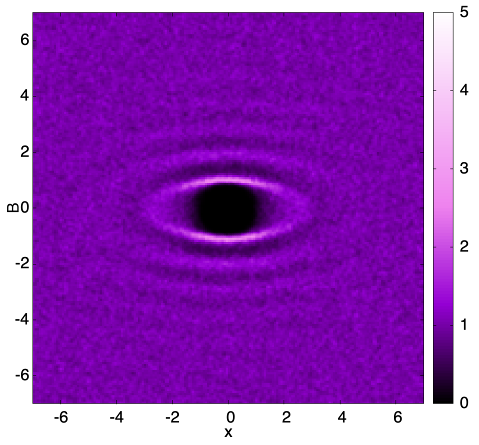

FIG. S8. Pair distribution function for a system of HEs at $\phi = 0.50$ with a magnetic field $\vec{B}$ directed along the y-axis.

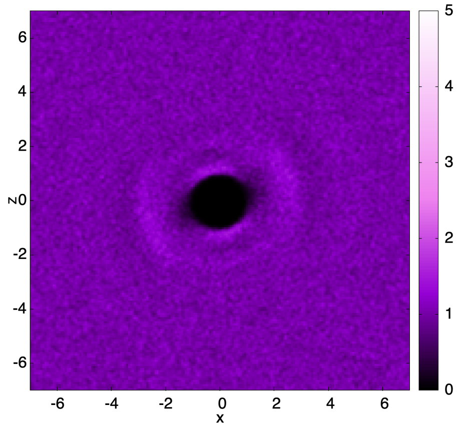

FIG. S9. Pair distribution function for a system of HEs at $\phi = 0.50$ with a magnetic field $\vec{B}$ along the y-axis.

C. Smectic and nematic order parameters

A convenient way to check the emergence of a smectic phase in our computer simulations is to calculate the smectic order parameter τ_1 defined as follows:

$$\tau_1 = \langle | \sum_i e^{i2\pi \vec{r}_i \cdot \hat{n}/d} | \rangle \tag{4}$$

where $\langle \ldots \rangle$ is an average over several independent configurations, $\vec{r}_i$ is the position of the i-th particle, $\hat{n}$ is the direction of the magnetic field and d is the thickness of the smectic

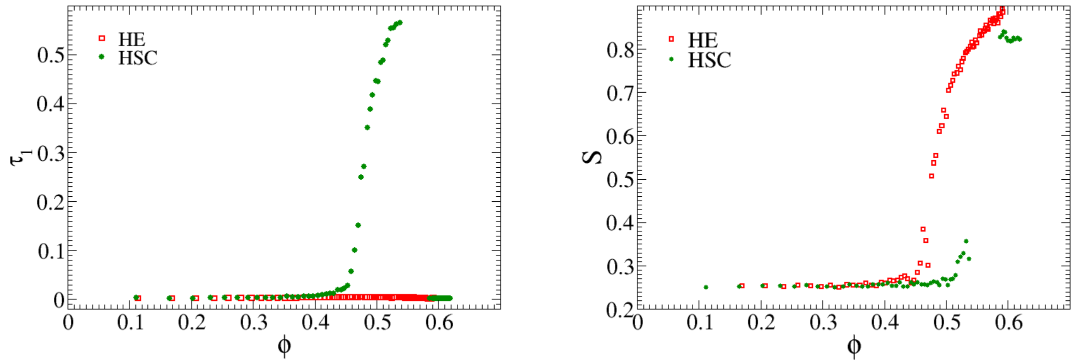

FIG. S10. Smectic (τ_1, left) and nematic (S, right) order parameters for HSCs and HEs.

layers. In order to compute τ_1 for each configuration we find the optimal value of d, i.e. the value which maximizes τ_1.

The values of τ_1 for both HSCs and HEs and for all pressures studied are shown in Fig. S10 (left). It can be seen that HSCs exhibit a range of volume fractions where a smectic layer ordering is present and which coincide with the green state points shown in Fig. 7(a) in the main test. On the contrary no evidence of layering emerges in the simulation of HEs.

We also calculated the nematic order parameter S related to the symmetry axis of HSC and HE, which is defined as the largest eigenvalue of the order tensor Q, whose components are:

$$Q_{\alpha\beta} = \frac{1}{N} \sum_i \frac{3}{2} \langle (\vec{u}_i)_\alpha (\vec{u}_i)_\beta \rangle \tag{5}$$

where $\alpha\beta \in \{x, y, z\}$ and the unit vector $(\mathbf{u}_i)_\alpha$ is the component α of the orientation (i.e. the symmetry axis) of particle i. First we note that since particles are aligned perpendicularly to the nematic field, if they are randomly oriented one has $S = 1/4$. Hence, below $\phi_0 < 0.45$ both symmetry axis of HSC and HE are randomly oriented onto the plance perpendicular to the magnetic field. Interestingly, for $\phi > \phi_0$ HSC starts exhibiting smectic layering but their orientations remain random, while orientations of HE starts aligning (see Fig. S10 (right)).

III. SIMULATIONS WITHOUT MAGNETIC FIELD

In order to demonstrate that the smectic phase observed in the simulations of HSCs is induced by the external magnetic field, we performed a NTV Monte Carlo simulation

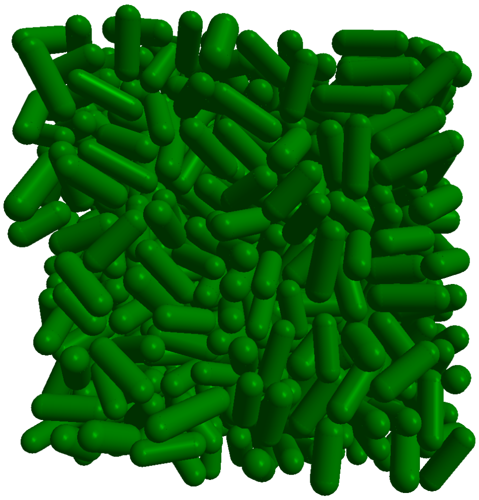

FIG. S11. Snapshot of a system of HSCs with $\phi = 0.50$ without an external magnetic field.

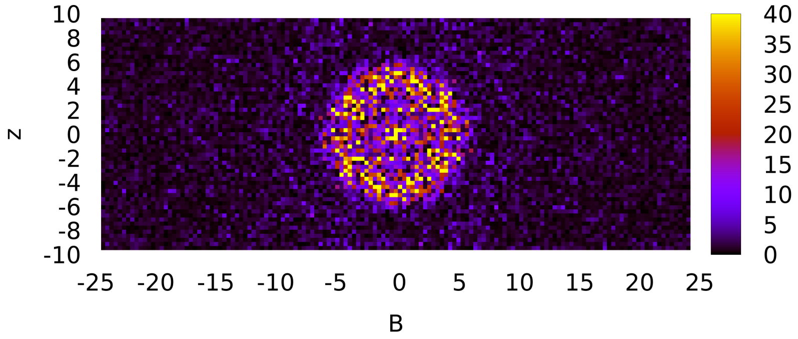

FIG. S12. Structure factor $S(0, q_y, q_z)$ of HSCs without a magnetic field at $\phi = 0.50$.

without the external magnetic field at $\phi = 0.50$ starting from a smectic configuration of HSCs. As shown in Fig. S11, the system is isotropic, thus proving that the smectic phase is an exquisite filed-induced effect.

A. Structure factor

To verify that by switching-off the external field an isotropic phase is obtained, we calculated also the static structure factor $S(0, q_y, q_z)$, which exhibits a typical isotropic pattern, as shown in Fig. S12.

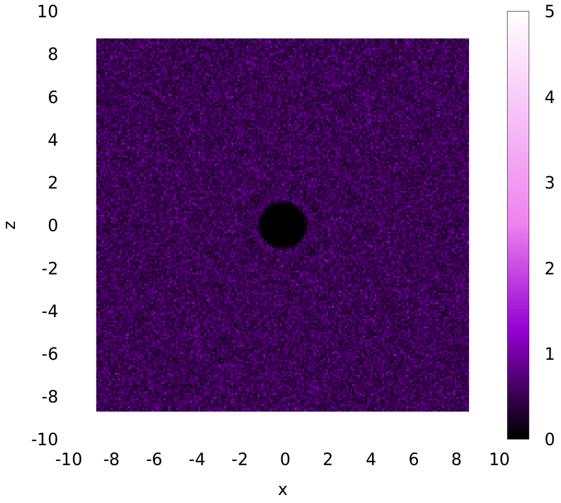

FIG. S13. Pair distribution function of HSCs without a magnetic field on the xz-plane at $\phi = 0.50$.

B. Pair distribution function

We also calculated the pair distribution function for HSCs with the magnetic field switched-off. As it can be seen in Figs. S13 and S14, the system is isotropic, i.e. long range spatial correlation between the particles are absent, thus confirming the field-induced nature of the smectic phase.

IV. STUDY OF HEMATITE-SILICA PARTICLE SHAPE

According to our computer simulations, the phase behavior under an external field is rather sensitive to particle shape, hence we carefully studied the shape of hematite-silica particles. We obtained the 2D contours (as set of 2D points) of particles with aspect ratio $\rho_1 = 2.82$ and $\rho_2 = 3.69$ from 2D TEM images (see Figs. 1(a) and 1(b) in the manuscript). The coordinates of these points will be measured in pixels which have to be meant as arbitrary units. Then, these contours were fitted by an ellipse ($\mathcal{F}_e$) and by the 2D curve which is obtained by projecting a spherocylinder onto a plane parallel to its symmetry axis ($\mathcal{F}_s$). Fits to $\mathcal{F}_e$ were performed by exploiting a recently developed and very efficient algorithm to find the roots of a quartic equation[4]. In the following, we will refer to latter curve also as "spherocylinder" for the sake of simplicity.

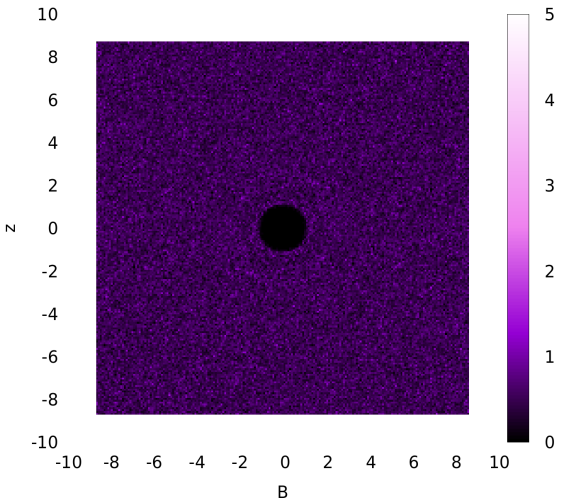

FIG. S14. Pair distribution function of HSCs without a magnetic field on the yz-plane at $\phi = 0.50$.

If $\mathcal{C}$ is the set of 2D points which forms the contour of a particle, then we can define the distance d between $\mathcal{C}$ and $\mathcal{F}_\alpha$, with $\alpha \in \{e, s\}$, as follows:

$$d \equiv \min_{\substack{(x_c,y_c)\\(x_{fit},y_{fit})}} \sqrt{(x_c - x_{fit})^2 + (y_c - y_{fit})^2} \tag{6}$$

where $(x_c, y_c) \in \mathcal{C}$ and (x_{fit}, y_{fit}) belongs either to $\mathcal{F}_e$ or $\mathcal{F}_s$. Note that, if $d = 0$ for a given particle, this means that this particle has a contour which is perfectly fitted by the chosen shape (i.e. either $\mathcal{F}_e$ or $\mathcal{F}_s$).

After having fitted all contours to both $\mathcal{F}_e$ and $\mathcal{F}_s$, we calculated according to Eq. 6 the distances d, from which we built the histograms shown in Fig. S16. Since the particles are not symmetric with respect to their major axis, we obtained an estimate of d from each of the two sides of each contours with respect of such axis (see Figs. S15(a) and S15(b)). It can be seen that the more elongated particles ($\rho = 3.69$) are more ellipsoidal, being the average value of d closer to 0 when particle contours are fitted by $\mathcal{F}_e$, than when they are fitted to $\mathcal{F}_s$ (see Fig. S16(a)). On the contrary, the contours corresponding to particles with $\rho = 2.82$ exhibit an hybrid nature since their average distances to $\mathcal{F}_e$ and to $\mathcal{F}_s$ have comparable values (see Fig. S16(b)).

To further characterize the shape of particles, starting from the contours $\mathcal{C}$ of all particles, for both $\rho = 3.69$ and $\rho = 2.82$, we built the "mean" contour which is shown in Fig. S17(a).

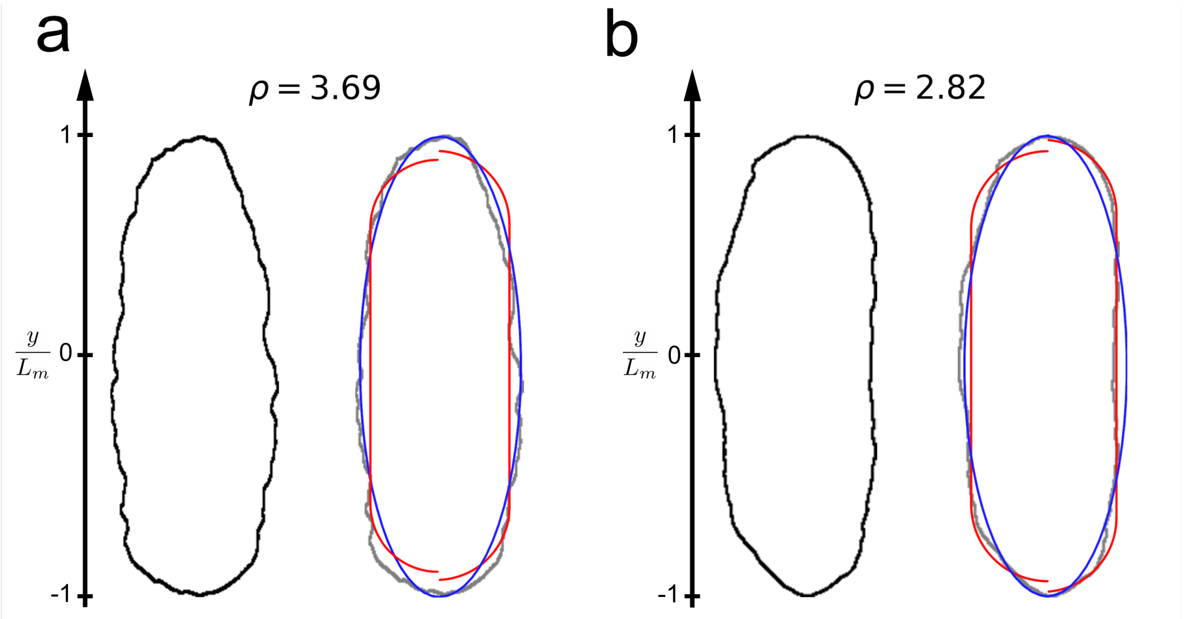

FIG. S15. Contours of particles with $\rho = 3.69$ (a) and $\rho = 2.82$ (b) has been fitted using an ellipse (in blue) and a spherocylinder (in red).

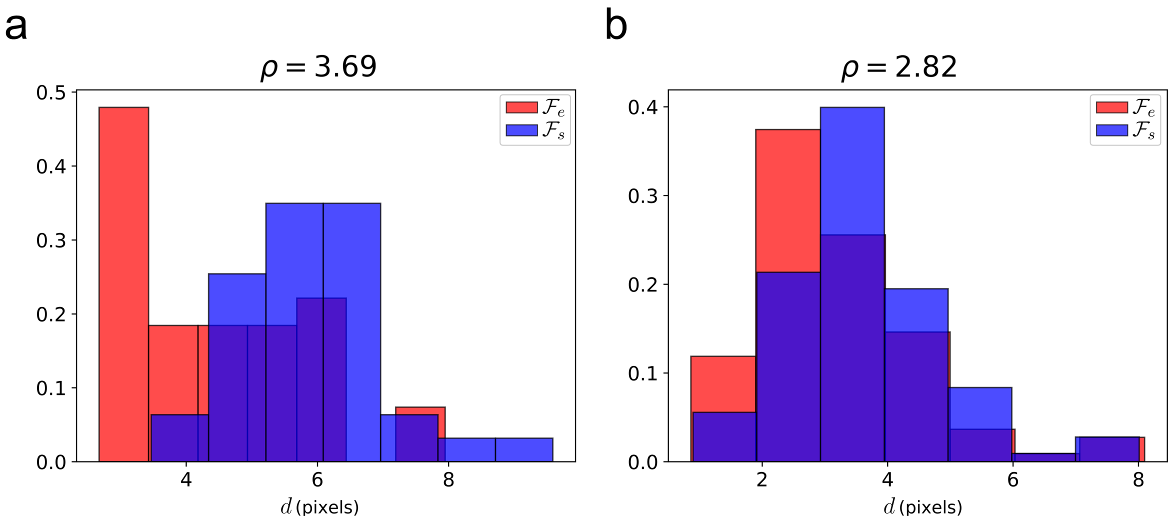

FIG. S16. Probability density of the distance d for both fitting shapes $\mathcal{F}_e$ (ellipse) and $\mathcal{F}_s$ (spherocylinder). (a) $\rho = 3.69$ and (b) $\rho = 2.82$.

Then, we fitted these mean contours to $\mathcal{F}_s$ and $\mathcal{F}_e$ and we calculated for each point belonging to the contour the quantity:

$$\widetilde{x}^2 = (x_c - x_{fit})^2 \tag{7}$$

Figure S17(b) shows $\widetilde{x}^2$ as a function of y_c/L_m (normalized position), where L_m is the length

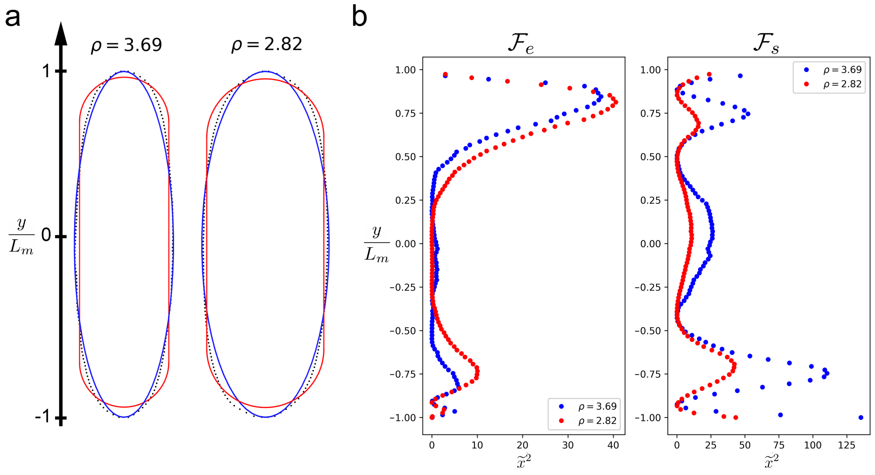

FIG. S17. (a) Mean contour of the particles for both elongations (black dots) together with the fits to $\mathcal{F}_e$ and $\mathcal{F}_s$ (blue and red curve respectively). (b) $\widetilde{x}^2$ as a function of y/L_m.

of the major axis of the mean contour.

First note that values of $\widetilde{x}^2$ for the fits to $\mathcal{F}_e$ are on average much smaller than the ones for the fits to $\mathcal{F}_s$, thus suggesting that an ellipsoidal shape better reproduces the particle contours. Nevertheless, while for the fit of the mean contour to an ellipse ($\mathcal{F}_e$) $\widetilde{d}^2$ is on average larger for the less elongated particles ($\rho = 2.82$) than for the more elongated ones (left panel of Fig. S17(b)), the opposite behavior is observed for the fit of the mean contour to $\mathcal{F}_s$ (right panel of Fig. S17(b)). Latter result highlights again the hybrid nature of particles with $\rho = 2.82$ which, to some extent, are more spherocylinder-like than the ones with $\rho = 3.69$.

To better elucidate the hybrid nature of particles with $\rho = 2.82$, in the following we will show that they possess on average a more cylinder-like midsection than the ones with $\rho = 3.69$. Hence, less elongated particles resemble more spherocylinders in their midsections, making them rather cylinder-like (i.e. “flat”).

We performed a fit of the contours of each particle to a straight line parallel to their major axis of length L (y-axis) in the interval $[-l/2, l/2]$ (with $l < L$) and we calculated the average distance d_f of the particle's contour from the fitting line over such interval. We repeated latter procedure for several values of l starting from $l = 2/L$ up to a value l_{max}, where $d_f > 5$ (in units of pixels). l_{max} quantifies the flatness of the midsection of the particles, in that the larger l_{max} and the flatter can be considered the midsection of particles.

The result of this analysis can be found in Fig. S18, where histograms of l_{max}/L values are

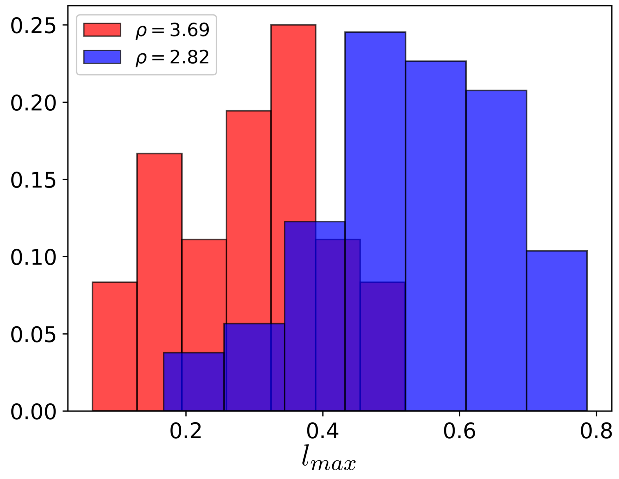

FIG. S18. Probability density for l_{max} for both aspect ratios $\rho = 3.69$ (red) and $\rho = 2.82$ (blue).

shown for both elongations. Since the histogram is peaked around a larger value of l_{max}/L for $\rho = 2.82$, one can conclude that less elongated particles have a larger cylinder-like (flat) midsection.

V. COMPARISON OF THE EXPERIMENTALLY OBTAINED INTENSITY PROFILES AND THE SIMULATED STATIC STRUCTURE FACTORS FOR THE NEMATIC AND SMECTIC PHASES

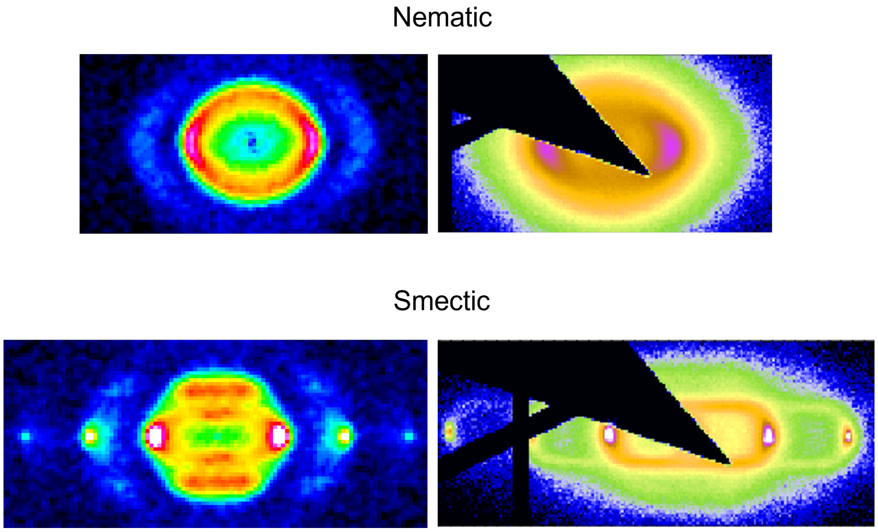

FIG. S19. Left panel corresponds to simulated static structure factor while the right panel corresponds to the experimentally obtained 2D intensity profiles for nematic and smectic phases.

REFERENCES

[1]A. Kuijk, D. V. Byelov, A. V. Petukhov, A. van Blaaderen, and A. Imhof, "Phase behavior of colloidal silica rods," Faraday discussions **159**, 181–199 (2012).

[2]D. V. Byelov, J.-M. Meijer, I. Snigireva, A. Snigirev, L. Rossi, E. van den Pol, A. Kuijk, A. Philipse, A. Imhof, A. van Blaaderen, *et al.*, "In situ hard x-ray microscopy of self-assembly in colloidal suspensions," RSC advances **3**, 15670–15677 (2013).

[3]J.-P. Hansen and I. R. McDonald, *Theory of Simple Liquids (Fourth Edition), With Applications to Soft Matter* (Academic Press, 2013).

[4]A. G. Orellana and C. D. Michele, "Algorithm 1010: Boosting efficiency in solving quartic equations with no compromise in accuracy," ACM Trans. Math. Softw. **46** (2020), 10.1145/3386241.
